# Identification of a Genetically Linked but Functionally Independent Two-Component System Important for Cell Division of the Rice Pathogen *Burkholderia glumae*

**DOI:** 10.3389/fmicb.2021.700333

**Published:** 2021-07-01

**Authors:** Joan Marunga, Eunhye Goo, Yongsung Kang, Ingyu Hwang

**Affiliations:** ^1^Department of Agricultural Biotechnology, Seoul National University, Seoul, South Korea; ^2^Research Institute of Agriculture and Life Sciences, Seoul National University, Seoul, South Korea

**Keywords:** *Burkholderia glumae*, two-component regulatory system, cell division, GluR, rice panicle blight

## Abstract

Bacterial two-component regulatory systems control the expression of sets of genes to coordinate physiological functions in response to environmental cues. Here, we report a genetically linked but functionally unpaired two-component system (TCS) comprising the sensor kinase GluS (BGLU_1G13350) and the response regulator GluR (BGLU_1G13360), which is critical for cell division in the rice pathogen *Burkholderia glumae* BGR1. The *gluR* null mutant, unlike the *gluS* mutant, formed filamentous cells in Lysogeny Broth medium and was sensitive to exposure to 42°C. Expression of genes responsible for cell division and cell-wall (*dcw*) biosynthesis in the *gluR* mutant was elevated at transcription levels compared with the wild type. GluR-His bound to the putative promoter regions of *ftsA* and *ftsZ* is involved in septum formation, indicating that repression of genes in the *dcw* cluster by GluR is critical for cell division in *B. glumae*. The *gluR* mutant did not form filamentous cells in M9 minimal medium, whereas exogenous addition of glutamine or glutamate to the medium induced filamentous cell formation. These results indicate that glutamine and glutamate influence GluR-mediated cell division in *B. glumae*, suggesting that GluR controls cell division of *B. glumae* in a nutrition-dependent manner. These findings provide insight into how the recognition of external signals by TCS affects the sophisticated molecular mechanisms involved in controlling bacterial cell division.

## Introduction

Two-component systems (TCSs) consisting of a sensor kinase and a cognate response regulator are common in bacteria (Gao and Stock, 2009). They are essential for the responses of bacteria to changes in environmental factors such as pH, osmotic pressure, antibiotics, and quorum-sensing (QS) signals (Gao and Stock, 2009). The sensor kinases are autophosphorylated after sensing an environmental stimulus, followed by phosphotransfer from the phosphorylated sensor kinases to the response regulators (Gao and Stock, 2009). The phosphorylated response regulators then undergo conformational changes to become active, thereby controlling the expression of target genes (Gao and Stock, 2009). The genes encoding sensor kinases and response regulators are often genetically linked in bacterial genomes and functionally paired (Stenson et al., 2005; Coutte et al., 2016). In addition to paired TCSs, sensor kinases and transcriptional regulators can crosstalk, thus modulating multiple biological processes in response to environmental signals irrespective of their genetic linkage (Hellingwerf, 2005; Yamamoto et al., 2005; Coutte et al., 2016; Chen et al., 2017).

We study the social behavior and host interactions of the rice bacterial pathogen *Burkholderia glumae*, the cause of rice panicle blight (Kim et al., 2004; Goo et al., 2015). A phytotoxin, toxoflavin, is the major virulence factor of *B. glumae* and exerts a toxic effect on photosynthetic organisms by generating radicals under light (Kim et al., 2004; Koh et al., 2011). The virulence-factor biosynthesis and motility of *B. glumae* are dependent on QS (Kim et al., 2004, 2007). As well as QS, we are interested in TCSs in *B. glumae* BGR1 because they coordinate and regulate the expression of genes critical for adaptation to stress, survival, fitness in the host, and virulence (Uhl and Miller, 1996; Perraud et al., 1998; Groisman, 2001; Bronner et al., 2004; Loui et al., 2009; Freeman et al., 2013; Yan et al., 2020). For instance, CpxAR of *Actinobacillus pleuropneumoniae* (Yan et al., 2020), ArcAB of *Escherichia coli* (Loui et al., 2009), and KdpDE (Freeman et al., 2013) and PhoPQ (Groisman, 2001) in a variety of bacterial taxa reportedly promote growth, fitness, and survival in the host. In addition, AgrAC, SsrAB, SaeRS, and ArlRS of *Staphylococcus aureus* and BvgAS of *Bordetella pertussis* are necessary for virulence (Uhl and Miller, 1996; Perraud et al., 1998; Bronner et al., 2004). Few studies have focused on TCSs in *B. glumae*, probably because of concern over repeating works on other pathogens. However, Karki et al. reported that the PidS/PidR TCS is essential for the pigmentation and virulence of *B. glumae* 411gr-6 (Karki et al., 2012).

In this study, we identified a TCS composed of the sensor kinase GluS and the response regulator GluR, which was critical for normal cell division in *B. glumae* BGR1. *gluR* and *gluS* were co-transcribed, but GluR functioned independently of GluS in normal cell division. We report that GluR regulates the gene cluster involved in cell division and cell wall (*dcw*) biosynthesis and conclude that external nutritional conditions modulate cell division in a TCS-dependent manner in *B. glumae*.

## Materials and Methods

### Bacterial Strains and Growth Conditions

The bacterial strains and plasmids used are listed in Supplementary Table 1. Unless stated otherwise, the strains were grown in Lysogeny Broth (LB) medium containing 0.1% (w/v) tryptone, 0.5% (w/v) yeast extract, 0.5% (w/v) sodium chloride, and 1.5% agar as required (Affymetrix, Cleveland, OH) with the appropriate antibiotics at 37°C. Antibiotics were used at the following concentrations: rifampicin, 100 μg/ml; ampicillin, 50 μg/ml; tetracycline, 10 μg/ml; kanamycin, 25 and 50 μg/ml; and spectinomycin, 50 μg/ml. 5-Bromo-4-chloro-3-indoyl-β-D-galactopyranoside (X-gal) was added at 40 μg/ml as necessary.

### DNA Manipulation and Sequencing

Basic DNA manipulation was conducted following standard protocols (Sambrook et al., 1989). Plasmid DNA from *E. coli* was isolated using the Biomedic Plasmid DNA Miniprep Kit (Ibiomedic, Bucheon, South Korea) following the manufacturer’s instructions. DNA sequencing was performed by Macrogen Inc. (Seoul, South Korea). The genetic information and gene IDs for DNA construction were obtained from the *B. glumae* BGR1 genome database (GenBank accession numbers: CP001503–CP001508; kropbase.snu.as.kr/cgi_bg.cg).

We used a previously constructed cosmid library of *B. glumae* BGR1 (Kim et al., 2004).

### Rescue *mini*-Tn*5*, Tn*3*-*gusA*, and Marker-Exchange Mutagenesis

With the use of *E. coli* S17-1 (pRescue *mini*-Tn*5*), random mutations were created in *B. glumae* BGR1 as described previously (De Lorenzo et al., 1990). Successful mutants were isolated by selection on LB agar containing kanamycin. The rescued *mini*-Tn*5* mutants were screened for phenotypic changes under the microscope. Following a previous method (Kwon and Ricke, 2000), the flanking regions were sequenced using the O-end primer (5′-GGTTTTCACCGTCATCACCG-3′), and the TCS genes were disrupted using the identified rescue *mini*-Tn*5* insertions. The selected mini-Tn*5rescue* mutant, RT271, was complemented by tri-parental mating (Figurski and Helinski, 1979) using pBGH1 plasmid to generate RT271C.

The pLAFR3 derivatives of pBGH1 carrying *gluR* (BGLU_1G13360) and *gluS* (BGLU_1G13350) were mutagenized using Tn*3*-*gusA* as described previously (Bonas et al., 1989). The Tn*3*-*gusA* insertion site and orientation in each mutant were mapped by restriction enzyme digestion analysis, and the plasmid was sequenced using the Tn*3*gus primer (5′-CCGGTCATCTGAGACCATTAAAAGA-3′). The plasmids carrying Tn*3*-*gusA* insertions were marker-exchanged into *B. glumae* BGR1 via tri-parental mating (Figurski and Helinski, 1979) to generate BGLUR133 and BGLUS35. All marker-exchange mutants were confirmed by Southern hybridization analysis.

We used the pBGH13 plasmid, a derivative of pBGH1, to complement the *gluR* mutant. First, pBGH1 DNA was digested with *Sca*I followed by ligation into pBluescript II SK (+). The resulting plasmid DNA was cut with *Bam*HI and *Hin*dIII followed by ligation into pLAFR3, resulting in pBGH13. The pBGH13 was introduced into BGLUR133 via tri-parental mating (Figurski and Helinski, 1979) to produce BGLUR133C.

### Bacterial Growth and Viability Assay

Overnight liquid cultures of the *B. glumae* strains were adjusted to an OD_600_ of 0.05 and subcultured into fresh LB medium. The cultures were incubated for 30 h at 37°C with shaking at 250 rpm. At 6-hour intervals, bacterial growth was assayed by spotting 10 μl of serial dilutions in triplicate on LB agar plates. Bacterial growth was expressed as log CFU/ml after 2 days of incubation at 37°C.

Cell viability was assayed using the LIVE/DEAD BacLight Bacterial Viability Kit, which contains SYTO 9 green-fluorescent nucleic acid stain and the red-fluorescent nucleic acid stain, and propidium iodide (Invitrogen, Carlsbad, CA, United States), following the manufacturer’s instructions. Fluorescence images were captured using a confocal laser scanning microscope (Leica SP8X, Wetzlar, Germany) at excitation/emission wavelengths of 483/490–540 and 535/890–680 nm for green and red fluorescence, respectively.

### Transmission Electron Microscopy

Bacterial cells were harvested from overnight cultures and prepared for observation by transmission electron microscopy (TEM), as reported previously (Kang et al., 2017). Electron micrographs were acquired using a JEM 1010 microscope (JEOL, Tokyo, Japan) with acceleration voltages of 180 and 100 kV from a LIBRA 120 energy-filtration microscope (Carl Zeiss, Oberkochen, Germany).

### Quantitative Reverse Transcription–Polymerase Chain Reaction

Total RNA was isolated from *B. glumae* BGR1, BGLUR133, and BGLUR133C using the RNeasy Mini Kit (Qiagen, Hilden, Germany) following the manufacturer’s instructions. Genomic DNA was removed using DNase I (Thermo Fisher Scientific, Vilnius, Lithuania). From 1 μg of RNA, reverse transcription for cDNA synthesis was performed at 42°C for 1 h with the Recombinant RNasin and M-MLV Reverse transcriptase following manufacturer’s instructions (Promega, Madison, WI, United States). With the use of specific primer sets (Supplementary Table 2), *ftsA*, *ftsB*, *ftsI*, *ftsK*, *ftsL*, *ftsQ*, *ftsW*, and *ftsZ* cDNAs were synthesized. Transcription levels were determined using SsoFast EvaGreen Supermix (Bio-Rad, Hercules, CA, United States) under the following conditions: 95°C for 30 s followed by 30 cycles of 95°C for 5 s and 55°C for 5 s. With the use of SensiFast SYBR No-ROX Kit (Bioline, Meridian Bioscience, Cincinnati, OH, United States), PCR was performed in triplicate, and gene expression values were normalized to 16S *rRNA* using Bio-Rad CFX Manager software.

### Growth and Viability of *Burkholderia glumae* Strains at 42°C

The *B. glumae* BGR1, TCS null mutants, and BGLUR133C strains were cultured overnight at 37°C, and the optical density at 600 nm (OD_600_) was adjusted to 0.05. The strains were incubated at 42°C with shaking at 250 rpm for 24 h in LB and M9 minimal media (6 g of Na_2_HPO_4_, 3 g of KH_2_PO_4_, 0.5 g of NaCl, and 1 g of NH_4_Cl in 1 L of deionized water containing 1 mM of MgSO_4_ and 0.1 mM of CaCl_2_, supplemented with 0.2% glucose), and the cell density was measured at 6-hour intervals.

### Environmental Stimuli Driving GluR Responses

We cultured the wild type, *gluR* mutant BGLUR133, and BGLUR133C in M9 minimal medium. To evaluate whether amino acids are required for GluR activity, M9 minimal medium was supplemented with 10% Bacto Casamino Acids (Becton, Dickson and Co., Franklin Lakes, NJ, United States) that comprise 20 essential amino acids. Individual amino acids (Sigma Aldrich, St. Louis, MI, United States) were analyzed at the concentrations in LB medium (Sezonov et al., 2007).

### Glutamate Utilization in *Burkholderia glumae*

Overnight liquid cultures of the wild-type BGR1 were adjusted to an optical density of OD_600_ of 0.05 and subcultured in LB medium for 24 h at 37°C with shaking at 250 rpm. At 3-hour intervals, the cultures were centrifuged (14,000 rpm, 4°C, 10 min), and the supernatants were collected. Glutamate analysis was carried out by liquid chromatography–mass spectrometry (LCMS-2000, Shimadzu, Kyoto, Japan) at the National Instrumentation Center and Environment Management (Seoul National University, Seoul, South Korea).

### Scanning Electron Microscopy

*B. glumae* strains cultured overnight in LB or M9 minimal medium with/without amino acids were harvested, fixed with Karnovsky’s fixative [2% glutaraldehyde, 2% paraformaldehyde in 0.05 M of sodium cacodylate buffer (pH 7.4)], and post-fixed with 1% sodium tetroxide in 0.1 M of sodium cacodylate buffer for 1 h at 4°C as described previously (Morris, 1965). Before imaging, the samples were coated with platinum at 10 mA for 270 s using a G20 Ion Sputter Coater (GSEM Co., Suwon, South Korea), and electron micrographs were acquired using a Carl Zeiss microscope (Auriga, Zeiss Germany).

### Electrophoresis Mobility Shift Assay

The coding region of *gluR* was amplified from BGR1 chromosomal DNA with the primers, gluR_Nde1-F and gluR_BamH1-R (Supplementary Table 2), and then cloned into the *Nde*I and *Bam*HI sites of pET21b (Invitrogen) to produce pGluR-His. GluR-His was overexpressed in *E. coli* strain BL21 (DE3) followed by purification using a Ni-NTA spin column in a buffer containing 50 mM of Tris–HCl (pH 8.0) and 100 mM of NaCl as described by the manufacturer (Qiagen). With primer sets ftsAp-F/R and ftsZ-F/R (Supplementary Table 2), the promoter regions of the putative GluR targets, *ftsAp* and *ftsZp*, respectively, were amplified. The resulting PCR products were labeled with biotin using LightShift Chemiluminescent Electrophoretic Mobility Shift Assay Kits, as described by the manufacturer (Pierce, Appleton, WI, United States). We used 329 bp upstream of *katE1* as a non-specific competitor DNA amplified using KatE1-F and KatE1-R primers (Supplementary Table 2). Purified GluR-His (0.75 μM) was incubated in binding buffer (10 mM of Tris–HCl (pH 7.5), 100 mM NaCl, and 5% (v/v) glycerol) containing 1 nM biotin-labeled DNA as described previously (Kim et al., 2007). For competition assays, unlabeled target DNA at 20-fold molar excess was added to each reaction with the labeled DNA. With the use of 4% (w/v) polyacrylamide gels, the reactions were separated and transferred to nitrocellulose membranes. The bands were detected using streptavidin/horseradish peroxidase-derived chemiluminescence kits, as described by the manufacturer (Pierce) and visualized using ChemiDoc XRS + and Image Lab Software (Bio-Rad).

### Statistical Analysis

All experiments were conducted in triplicate with the appropriate controls. One-way analysis of variance (ANOVA) followed by Tukey’s honestly significant difference *post hoc* analysis in SPSS software (ver. 25 × 86-x64; IBM Corp., Armonk, NY, United States) were conducted to detect significant differences. A value of *p* < 0.05 was considered indicative of statistical significance.

### Results Figure Preparation

All the figures presented in this manuscript were prepared using Adobe illustrator 2020 v. 24.3, available at: https://dobe.com/products/illustrator.

## Results

### Identification of a Two-Component System Critical for Normal Cell Division of *Burkholderia glumae* BGR1

**FIGURE 1:**
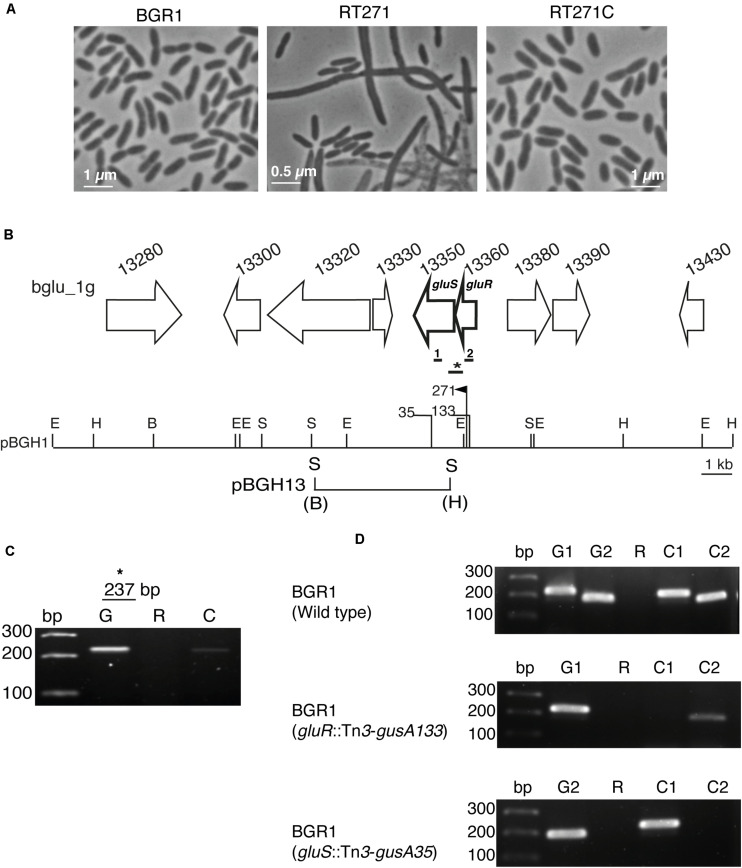
The GluS-GluR two-component system (TCS) of *Burkholderia glumae* BGR1. **(A)** Microscopic observation of the cell morphology of BGR1 (wild type), RT271 (BGR1, *gluR*:mini-Tn*5rescue*), and mutant complemented RT271C (BGR1, *gluR*:mini-Tn*5rescue* carrying pBGH1) strains in Lysogeny Broth (LB) medium. **(B)** Organization scheme of the GluS sensor kinase and GluR response regulator in pBGH1. Vertical bars above the restriction map indicate the position of the Tn*5* (271) and Tn*3*-*gusA* insertions (35 and 133). E, *Eco*RI; H, *Hin*dIII; B, *Bam*HI; S, *Sac*I. The map below pBGH1 represents pBGH13 plasmid of a 6.3-kb fragment containing *gluS* and *gluR* genes. **(C)** RT-PCR analysis showing that *gluS* and *gluR* genes are co-transcribed in BGR1. Primers were designed to amplify a 237-bp (*) product encompassing the *gluR* and *gluS* genes in the wild type. Lane G, PCR product using genomic DNA as a template; Lane R, PCR product using RNA as a template; Lane C, PCR product using cDNA as a template. **(D)** No polar effect resulting from Tn*3*-*gusA* insertion. Lane bp, marker; Lane G1, PCR product from *gluR* chromosomal DNA as a template; Lane G2, PCR product from *gluS* chromosomal DNA as a template; Lane R, PCR product from total RNA as a template; Lane C1, PCR product from *gluR* cDNA as a template; Lane C2, PCR product from *gluS* cDNA as a template. The thick bars below the gene map indicate the position that was amplified for cDNA synthesis. Primers were designed to amplify 200 bp (1) and 216 bp (2) product of *gluS* and *gluR*, respectively. Full gel images are presented in Supplementary Figures 6, 7.

To identify a key TCS important for normal cell division of *B. glumae* BGR1, we first mutagenized it with mini-Tn*5* and examined the morphology of the mutants. The mutant RT271 formed filamentous cells when grown in LB medium (Figure 1A); and its respective mutant complementation, RT271C, restored the rod-shaped cells similar to those in the wild-type BGR1 (Figure 1A). To determine the insertion site of mini-Tn*5* in the RT271 mutant, a mini-Tn*5* insertion along with flanking sequences was rescued by digestion of its genomic DNA with *Eco*RI, self-ligation, and transformation into *E. coli* DH5α. Flanking sequences of mini-Tn*5* from the rescued plasmid pRT271E revealed that an annotated gene BGLU_1G13360 had an insertional mutation (Figure 1B). This gene, *gluR*, encoded a 27.7-kDa protein that exhibited 99.6% similarity to known OmpR-type response regulators such as BURPS305_7006 in *Burkholderia pseudomallei* 305, RisA (BMA10247_1253) in *Burkholderia mallei* NCTC 10247, and BCENMCO3_1962 of *Burkholderia cenocepacia* MCO-3 (Supplementary Figure 1A). Downstream of *gluR* was a putative sensor kinase, *gluS* (BGLU_1G13350) (Figure 1B) that showed 96.7%, 94.1%, and 90.0% identities with known sensor kinases such as Envz1 (BGL_1C23830) in *Burkholderia plantarii*, BGLA_1G24110 in *Burkholderia gladioli* BSR3, and RisS (BMA1486) in *B*. *mallei* ATCC 23344, respectively (Supplementary Figure 1B).

Due to the proximity of *gluS* and *gluR* in the BGR1 genome, we reasoned that these two genes might be co-transcribed into a polycistronic RNA. Therefore, we performed reverse transcription–polymerase chain reaction (RT-PCR) with specific primers, GluSR-F/R (Figure 1B and Supplementary Table 2). We found that *gluR* and *gluS* were indeed co-transcribed (Figure 1C). We next mutagenized pBGH1, a cosmid carrying *gluS* and *gluR*, with Tn*3*-*gusA* to generate mutants of *gluR* and *gluS* followed by marker exchange into *B. glumae* BGR1, resulting in BGLUR133 (BGR1 *gluR*:Tn*3*-*gusA133*) and BGLUS35 (BGR1 *gluS*:Tn*3*-*gusA35*) (Figure 1B). In the *gluR* mutant, the expression of *gluS* was decreased, suggesting the possibility of polar effects due to the transposon insertion in *gluR* (Figure 1D).

### Aberrant Cell Division Due to a Mutation in *gluR*

**FIGURE 2:**
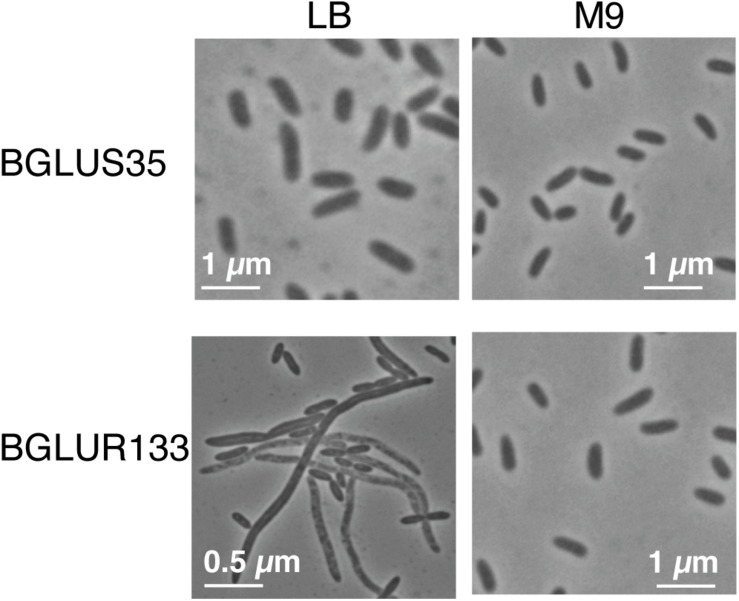
Tn*3*-*gusA* mutations in *gluR* resulted in nutrient-dependent cell filamentation. In Lysogeny Broth (LB) medium, the *gluR* mutant BGLUR133 formed filamentous cells, but a normal rod-shaped cell morphology was observed in M9 minimal medium. No morphological defects were observed in the *gluS* mutant BGLUS35 in the different culture media.

**FIGURE 3:**
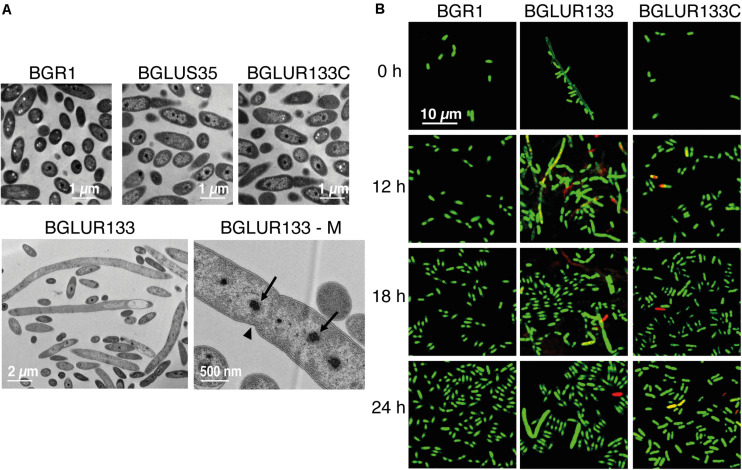
The *gluR* mutant forms a heterogeneous population of viable filamentous and normal rod-shaped cells. **(A)** The indicated bacterial strains were grown to the early stationary phase, and the morphological phenotypes of ultrathin sections were observed by transmission electron microscopy (TEM). BGLUR133-M shows that the filamentous cells formed by the *gluR* mutant contained multiple nuclei (arrows) with indents (arrowhead) along the cell wall, symbolizing failed septum formation. **(B)** Cell viability of the wild type, BGLUR133, and complemented BGLUR133C strains assessed by combination staining with propidium iodide (PI) and SYTO 9 green. Fluorescence images were obtained by confocal laser scanning microscopy. Dead cells stained with PI are red, and SYTO 9-stained viable cells are green.

To determine whether the insertion of Tn*3*-*gusA* in *gluR* or *gluS* conferred a similar cell morphology to the RT217 mutant, we observed the morphology of the *gluR* and *gluS* mutants under a light microscope. The *gluR* mutant BGLUR133 showed extensive filamentous cells in LB medium (Figure 2), consistent with the initial phenotype of the *gluR*:min-Tn*5* mutant RT271 in LB (Figure 1A). However, the *gluS* mutant BGLUS35 formed normal cells in LB medium (Figure 2), implying that the polar effect seen in Figure 1D was not significant. The *gluR* mutant BGLUR133 maintained a normal rod-shaped cell morphology similar to that of the *gluS* mutant BGLUS35 in M9 minimal medium supplemented with glucose (Figure 2). Evaluating the expression of *gluS* and *gluR* in LB and M9 minimal medium showed that *gluR* was more abundant in LB than M9 medium, while *gluS* showed the opposite pattern (Supplementary Figures 2A,B), suggesting that nutrient conditions may differentially regulate the two genes. TEM of ultrathin sections of the *gluR* mutant BGLUR133 revealed characteristic features of filamentous cells with multiple nuclei and indents along the cell membrane at points where the septum would have formed to separate dividing cells (Figure 3A). The genetically complemented strain of the *gluR* mutant BGLUR133 with pBGH13, BGLUR133C, had morphologically uniform rod-shaped cells (Figure 3A). The growth of the *gluR* mutant BGLUR133 and the wild-type BGR1 for 30 h in LB medium at 37°C was similar (Supplementary Figure 3 and Figure 3B). Although filamentous cells of the *gluR* mutant BGLUR133 remained viable for 30 h, their abundance decreased after 18 h, as observed under the microscope (Figure 3B).

### Direct Control of Genes Involved in Cell Division by GluR

**FIGURE 4:**
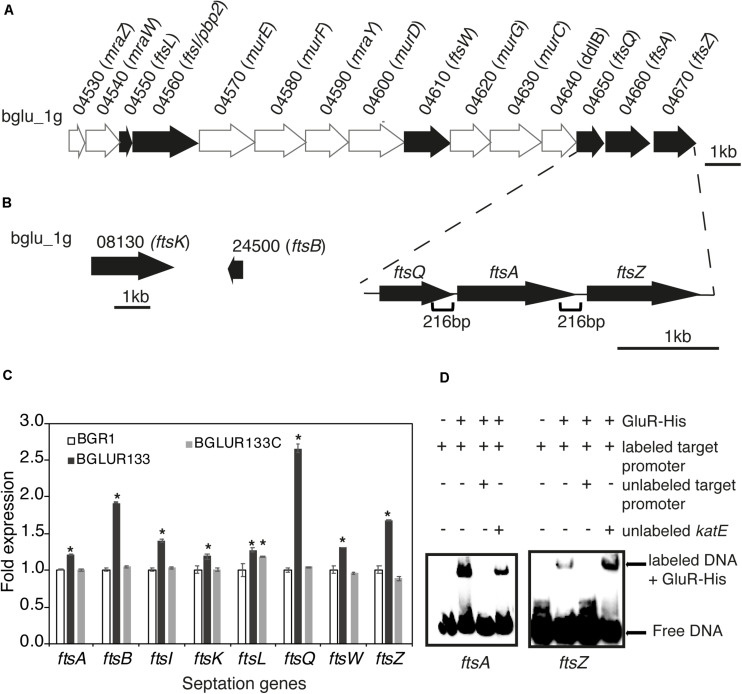
GluR represses cell division and septation genes in *Burkholderia glumae*. **(A)** Genetic organization of the *dcw* cluster in BGR1. Dark arrows represent genes involved in septation during cell division, and light arrows are genes involved in cell-wall synthesis or with no known function. The section below the gene map indicates the positions and size of the respective putative promoters in this study. **(B)** Gene maps of additional cell-division genes outside the *dcw* cluster. **(C)** Expression levels of eight cell-division genes in the wild type (BGR1), *gluR* mutant, and complemented BGLUR133C strains compared by qRT-PCR. mRNA levels were normalized to 16S *rRNA*, and the fold expressions are relative to those of the wild type. Data are means ± standard error (SE) of triplicates; statistical analysis was performed by one-way ANOVA/Tukey’s correlation for multiple comparisons. *p < 0.05; [*F* (48,71) = 1,536.273; *p* = 0.00]. **(D)** Electrophoresis mobility shift assay (EMSA) showed direct control of *ftsA* and *ftsZ* by GluR-His binding to the respective putative promoter regions; 0.75 μM of GluR-His, 1 nM of labeled target DNA, 1 nM of unlabeled *katE* non-competitor DNA, and 20 nM of unlabeled target promoter DNA were used for EMSA. Full blot images with multiple contrasts are shown in Supplementary Figure 8.

Because TEM suggested the involvement of GluR in cell division, we determined whether GluR influences the expression of genes in the *dcw* cluster involved in cell division. In *B. glumae*, there were 15 annotated genes: e.g., *ftsA*, *ftsI*, *ftsL*, *ftsQ*, *ftsW*, and *ftsZ*, in the *dcw* cluster and *ftsB* and *ftsK* in other regions (Figures 4A,B). The expression levels of *ftsA*, *ftsB*, *ftsI*, *ftsK*, *ftsL*, *ftsQ*, *ftsW*, and *ftsZ* in the *gluR* mutant BGLUR133 were significantly increased compared with those in the wild-type BGR1 (Figure 4C). The expression levels of the eight genes in BGLUR133C were similar to those in the wild type (Figure 4C). To determine whether GluR directly controls their expression, we performed electrophoresis mobility shift assays (EMSA) on the putative promoter regions of *ftsA* and *ftsZ* and purified His-tagged GluR (GluR-His). The binding of GluR-His to the putative promoter regions of *ftsA* and *ftsZ* confirmed that GluR-His directly represses the expression of cell division genes in *B. glumae* (Figure 4D). In the upstream regions of f*tsA* and *ftsZ*, we found a conserved inverted repeat sequence, indicating a potential GluR binding site (Supplementary Figure 4).

### Influence of Glutamate and Glutamine on GluR-Mediated Control of Cell Division

**FIGURE 5:**
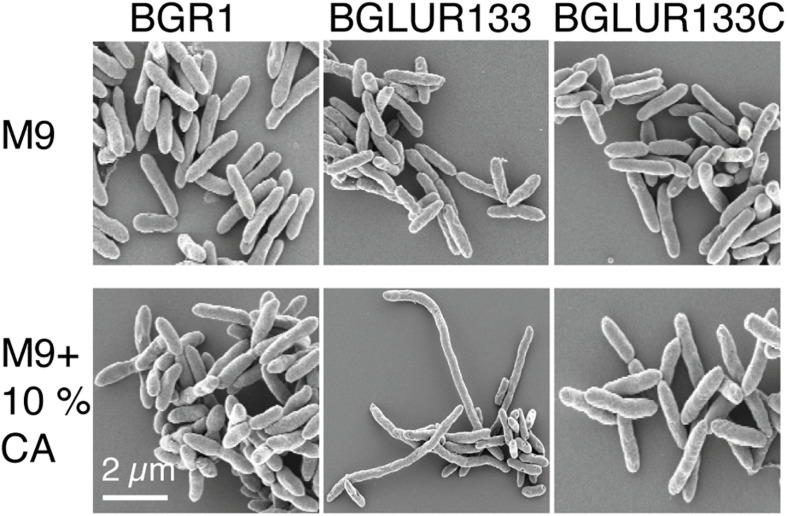
Extracellular amino acids promote filamentation in response to GluR mutation. The indicated bacterial strains were cultured overnight in M9 minimal medium with or without 10% casamino acids (CA) and processed for SEM analysis, and their morphology was observed using a Carl Zeiss GmbH Auriga microscope.

**FIGURE 6:**
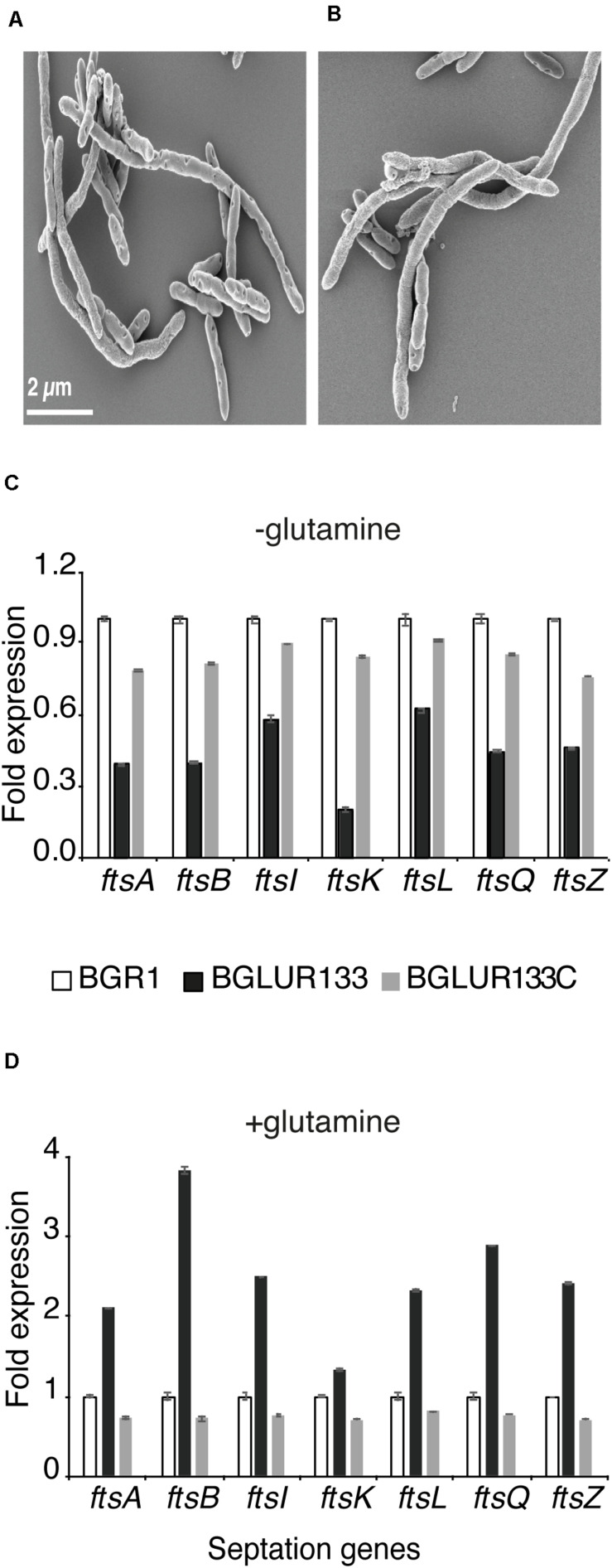
Extracellular glutamine and glutamate are required for GluR-mediated cell division. The *gluR* mutant BGLUR133 formed filamentous cells in glutamine **(A)** and glutamate **(B)**-rich M9 minimal medium. **(C,D)** Differences in the expression levels of septation genes in plain M9 **(C)** and glutamine-rich M9 minimal medium **(D)**, analyzed by qRT-PCR using the wild-type BGR1 as the baseline. Data are means ± standard error (SE) of triplicates.

Because the *gluR* mutant BGLUR133 formed filamentous cells in LB medium but not in M9 minimal medium, we reasoned that the amino acids in LB medium might be the cause of filamentous cell formation. Therefore, we added 10% casamino acids to M9 minimal medium containing glucose to evaluate their influence on the morphology of the *gluR* mutant BGLUR133. Adding casamino acids to M9 minimal medium transformed the morphologically normal cells of the *gluR* mutant BGLUR133 into filamentous cells (Figure 5). To identify the amino acids(s) responsible for triggering filamentous cells in the *gluR* mutant BGLUR133, 20 amino acids were individually added to M9 minimal medium. Of the 20 amino acids, only glutamine and glutamate individually triggered cells of the *gluR* mutant BGLUR133 to become filamentous in M9 minimal medium (Figures 6A,B). These results suggested that glutamine and glutamate play a role in *gluR*-mediated cell division in *B. glumae*.

Because environmental glutamine affected the cell morphology of the *gluR* mutant BGLUR133 in M9 minimal medium, we examined the expression levels of seven *fts* genes in M9 minimal medium with or without glutamine. In the absence of glutamine, the expression levels of the seven *fts* genes were significantly lower in the *gluR* mutant BGLUR133 than in the wild type, or the BGLUR133C complemented strain (Figure 6C). However, the addition of glutamine to M9 minimal medium increased the expression levels of the seven *fts* genes in the *gluR* mutant BGLUR133 (Figure 6D).

### Heat Sensitivity Due to Altered *fts* Gene Expression in the *gluR* Mutant

**FIGURE 7:**
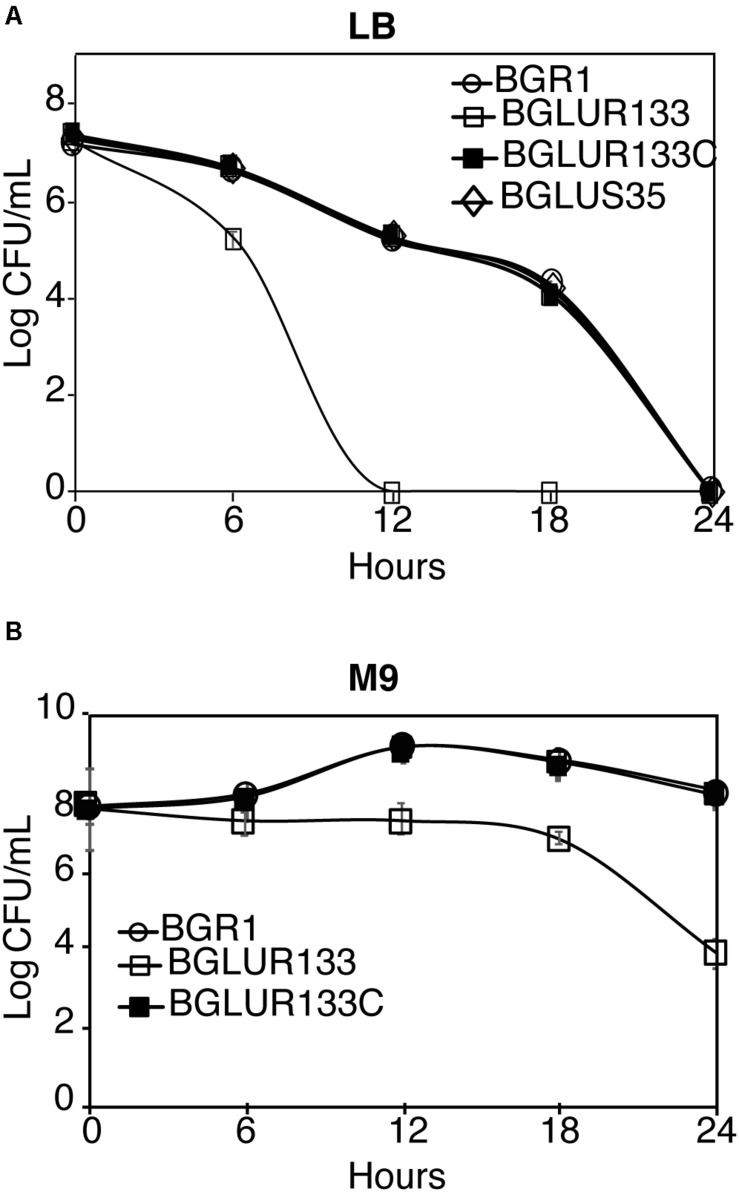
Exponential population decline at 42°C as a result of mutations in GluR. At 6-h intervals, the indicated strains’ population densities in Lysogeny Broth (LB) medium **(A)** and M9 medium **(B)** were quantified by colony-forming unit (CFU) counting, and the results are expressed as log CFU/ml. Data are means ± standard error (SE) of triplicates.

Because *fts* genes were identified in a temperature-sensitive filamenting mutant, we assessed whether the filamenting *gluR* mutant BGLUR133 is heat sensitive. Despite having no effect at 37°C (Supplementary Figure 3), the number of cells of the *gluR* mutant BGLUR133 decreased significantly after 6 h at 42°C, and they were entirely non-viable after 12 h in LB medium (Figure 7A). The wild-type BGR1, the *gluS* mutant BGLUS35, and the complemented strain BGLUR133C showed no growth but prolonged survival at 42°C (Figure 7A). In M9 minimal medium at 42°C, the *gluR* mutant BGLUR133 retained viability for 18 h and subsequently lost viability (Figure 7B). By contrast, the wild-type BGR1 and the complemented strain BGLUR133C increased in cell number during the static period of *gluR* mutant BGLUR133 in M9 medium (Figure 7B).

## Discussion

In addition to QS systems, pathogens also respond to environmental factors using TCSs. Here, we investigated the response regulator, GluR, which is crucial for normal cell division in *B. glumae*. Although *gluR* and *gluS* were co-transcribed, GluS may not be a *bona fide* counterpart of GluR because a mutation in *gluS* did not affect the normal cell division of *B. glumae*. Such a genetically linked but functionally independent TCS was reported for *risS* and *risA*, which encode a sensor kinase and a response regulator, respectively, in *B*. *pertussis* (Stenson et al., 2005). *risS* and *risA* were genetically linked but functionally independent (Stenson et al., 2005). Phosphorylation of RisA was mediated by crosstalk with a non-operonic histidine kinase, RisK (Chen et al., 2017). Therefore, an as-yet-unidentified sensor kinase may be responsible for the phosphorylation of GluR in *B. glumae*.

In rod-shaped bacteria such as *B. glumae*, cell division involves ingrowth of the cell wall and membrane, forming a septum between two replicated chromosomes (Harry, 2001). To ensure equal partitioning of chromosomes into daughter cells, the expression of genes involved in cell division must be properly regulated (Dai and Lutkenhaus, 1992; Harry, 2001). In most bacteria, cell division and cell-wall synthesis are regulated by a series of genes in the *dcw* cluster (Dai and Lutkenhaus, 1992). Within bacterial groups of the same class and cell shape, the order and regulation of genes in the *dcw* cluster are highly conserved (Pilhofer et al., 2008). Therefore, it was not surprising that in *B. glumae*, the *dcw* cluster displayed significant similarities to that of *E. coli* (Vicente et al., 1998). Pioneer studies of the *dcw* cluster genes in *E. coli* spotlighted *ftsZ* as the key element in cell division (Bi and Lutkenhaus, 1991; De Boer et al., 1992). It was later noted that FtsZ is not sufficient to drive septation, leading to the discovery of, for instance, *ftsA*, *ftsQ*, and *ftsI* (Vicente et al., 1998). In a hierarchical order initiated by the assembly of FtsZ at the division site, the *dcw* proteins are coordinately involved in cell division and the synthesis of the peptidoglycan precursors (Vicente et al., 1998; Lutkenhaus and Du, 2017).

The mechanisms of regulation of the *dcw* cluster are unclear, despite the presence therein of several regulatory elements, e.g., internal promoters, transcript stabilizers, and protein ratios (Vicente et al., 1998; Francis et al., 2000). Studies on the control of cell division have concentrated on FtsZ. Multiple promoter regions have been reported upstream of *ftsZ* in the *dcw* cluster, indicating regulation at the transcriptional level (Vicente et al., 1998; Francis et al., 2000; Margolin, 2000). We found that GluR binds to the upstream promoter regions of *ftsZ* and *ftsA* located in the *ftsA* and *ftsQ* coding regions, respectively. A conserved inverted repeat sequence was also found in the upstream sequences of *ftsA* and *ftsZ*. This suggested a possible GluR binding sequence, albeit mutational analysis of the putative promoter regions of both genes and DNase footprinting experiments are needed to validate it. Unlike positive regulators in *E. coli*, such as SdiA (Sitnikov et al., 1996), the phase-specific sigma factor (Ballesteros et al., 1998), and RcsB (Carballès et al., 1999), GluR negatively regulates cell division in *B. glumae*. Biased expression of the *dcw* cluster genes resulting from a mutation in *gluR*-induced aberrant cell division suggested that the GluR-controlled expression of *dcw* cluster genes was essential for normal cell division in *B. glumae*. Similar findings have been reported in other bacteria species, such as *E. coli*, where high levels of *dcw* genes expression impeded cell division, as seen by the production of filamentous cells in some cases (Dai and Lutkenhaus, 1992).

Because LB medium is rich in amino acids, and filamentation of the *gluR* mutant was facilitated by supplementation of extracellular glutamine or glutamate in M9 medium, the glutamine-dependent filamentous cell formation at an early stage of growth in LB was explicable. However, the number of filamentous cells of the *gluR* mutant BGLUR133 decreased over time, possibly as a result of depletion of amino acids, including glutamine and glutamate, 12 h after incubation (Supplementary Figure 5). It is clear that GluR and glutamine/glutamate are involved in normal cell division of *B*. *glumae*; however, it is not conclusive how these and other factors work together for the *gluR* mutant phenotypes in M9 minimal medium. Extracellular glutamine and glutamate reportedly alter the expression of genes involved in cell division and cell-wall synthesis of *Bacillus subtilis* (Ye et al., 2009). Beuria et al. reported an increased FtsZ polymerization rate and extent in *E. coli* that resulted from extracellular glutamine (Beuria et al., 2003). It was noted that FtsZ showed optimal polymerization as large, bundled filamentous structures in *E. coli* in the presence of 1 M of glutamine (Beuria et al., 2003). Interestingly, FtsZ polymers formed in the absence of glutamine were 9-fold less stable than those in its presence, emphasizing the roles of these amino acids in the stability of FtsZ polymers (Beuria et al., 2003).

Connections between TCS and glutamine metabolism have been reported in other bacteria. For example, GlnK-GlnL of *B. subtilis* (Satomura et al., 2005), GluR-GluK of *Streptomyces coelicolor* (Li et al., 2017), and AauR-AauS of *Pseudomonas putida* (Sonawane et al., 2006) reportedly sense and control glutamate uptake. In other bacteria, the TCSs involved in glutamine sensing and uptake are located close to the glutamine ABC transporter (Satomura et al., 2005; Sonawane et al., 2006; Li et al., 2017). However, GluR is not likely to be involved in glutamine uptake because we reported that GltI is responsible for glutamine uptake in *B. glumae* (Kang et al., 2017). A *bona fide* sensor kinase responsible for glutamine sensing and GluR phosphorylation is yet to be identified in *B. glumae*.

Physiological experiments in *E. coli* demonstrated that mutated septation genes resulted in elongated cells and an exponential population decrease at high temperatures, giving the mutants the name filamentous temperature sensitive (Ricard and Hirota, 1973). While we did not specifically modify the septation genes of *B. glumae* in the *gluR* mutant BGLUR133, we observed identical phenotypes of cell elongation and sensitivity to heat treatment at 42°C as in *E. coli* with mutated septation genes (Ricard and Hirota, 1973). These findings further support the hypothesis that GluR is crucial for cell division and an optimum gene expression profile. Taken together, our findings indicate that GluR is key for maintaining the gene expression profile required for glutamine- or glutamate-dependent control of cell division in *B. glumae* BGR1.

## Data Availability Statement

The original contributions presented in the study are included in the article/Supplementary Material, further inquiries can be directed to the corresponding author/s.

## Author Contributions

JM and IH designed the experiments. JM and EG performed the experiments. JM, EG, YK, and IH analyzed the data. JM and IH contributed reagents/materials, and analysis tools, and wrote the manuscript. All authors contributed to the article and approved the submitted version.

## Conflict of Interest

The authors declare that the research was conducted in the absence of any commercial or financial relationships that could be construed as a potential conflict of interest.
