# Supplementary material for: Identification of a Genetically Linked but Functionally Independent Two-Component System Important for Cell Division of the Rice Pathogen Burkholderia glumae
Source: Front Microbiol. 2021 Jul 1;12:700333. doi: 10.3389/fmicb.2021.700333 (PMC8281045; doi:10.3389/fmicb.2021.700333)
Supplement: Supplementary file 1 [file Data_Sheet_1.pdf]

Supplementary Material

Figure S1. Similarities among two-component system protein sequences computed by ClustalW multiple sequence alignment using the BioEdit sequence alignment editor. (**A**) Comparison between the GluR response regulator of *B. glumae* BGR1 (BGLU_1G13360), BURPS305_7006 of *B. pseudomallei* 305, RisA BMA10247_1253 of *B. mallei* NCTC 10247, and BCENMCO3_1962 of *B. cenocepacia* MCO-3. (**B**) Comparison between the GluS sensor kinase of *B. glumae* BGR1 (BGLU_1G13350), Envz1 BGL_1C23830 of *B. plantarii*, BGLA_1G24110 of *B. gladioli* BSR3, and RisS BMA1486 of *B. mallei* ATCC 23344.

(A) **Two-component system Response regulators**

```
BGLU_1G13360   MPPMETKNPS KILVVDDDPR LRDLLRRYLG EQGFNVYVAE NATAMNKLWV RERFDLLVLD
BURPS305_7006  MPLMETKNPS KILVVDDDPR LRDLLRRYLG EQGFNVYVAE NATAMNKLWV RERFDLLVLD
BMA10247_1253  MPLMETKNPS KILVVDDDPR LRDLLRRYLG EQGFNVYVAE NATAMNKLWV RERFDLLVLD
BCENMCO3_1962  MPIMETKNPS KILVVDDDPR LRDLLRRYLG EQGFNVYVAE NATAMNKLWV RERFDLLVLD

               ....|....| ....|....| ....|....| ....|....| ....|....| ....|....|
                     65         75         85         95        105        115
BGLU_1G13360   LMLPGEDGLS ICRRLRGSND RTPIIMLTAK GEDVDRIVGL EMGADDYLPK PFNPRELVAR
BURPS305_7006  LMLPGEDGLS ICRRLRGSND RTPIIMLTAK GEDVDRIVGL EMGADDYLPK PFNPRELVAR
BMA10247_1253  LMLPGEDGLS ICRRLRGSND RTPIIMLTAK GEDVDRIVGL EMGADDYLPK PFNPRELVAR
BCENMCO3_1962  LMLPGEDGLS ICRRLRGSND RTPIIMLTAK GEDVDRIVGL EMGADDYLPK PFNPRELVAR

               ....
```

(B) **Two-component system Sensor kinases**

```
BGLU_IG13350  MRIDRRLLTL VFGGLFWRTF LLIALLIAVS LAAWFQSFRV IEREPRAQRV ALQLVAVVKL
BGL_1C23830   MRIDRRLLTL VFGGLFWRTF LLIALLIAVS LAAWFQSFRV IEREPRAQRV ALQLVAVVKL
BGLA_1G24110  MRIDRRLLTL VFGGLFWRTF LLIALLIAVS LAAWFQSFRV IEREPRAQRV ALQLVAVVKL
BMA1486       MRIDRRLLTL VFGGLFWRTF LLIALLIAVS LAAWFQSFRV IEREPRAQRV ALQLVAIVKL

              ....|....| ....|....| ....|....| ....|....| ....|....| ....|....|
                    65         75         85         95        105        115
BGLU_IG13350  TRTALLYSDP DLRRALLQDL ESNEGVRVYP RESTDKFKLQ PDESLNRLIE HDIRSRLGDD
BGL_1C23830   TRTALLYSDP DLRRALLQDL ESNEGVRVYP RETTDKFKLQ PDESLNRLIE HDIRSRLGDD
BGLA_1G24110  TRTALLYSDP DLRRALLQDL ESNEGVRVYP RETTDKFKLQ PDESLNRLIE HDIRSRLGDD
BMA1486       TRTALLYSDP DLRRALLQDL ESNEGVRVYP REKTDKFKLQ PDESVNRLIE HDIRSRLGDD

              ...
```

Figure S2. The expression of *gluR* and *gluS* in different culture media. qPCR analysis of *gluR* **(A)** and *gluS* **(B)** expression in BGR1 cultured in LB and M9 minimal medium was performed and normalized to 16S *rRNA*.

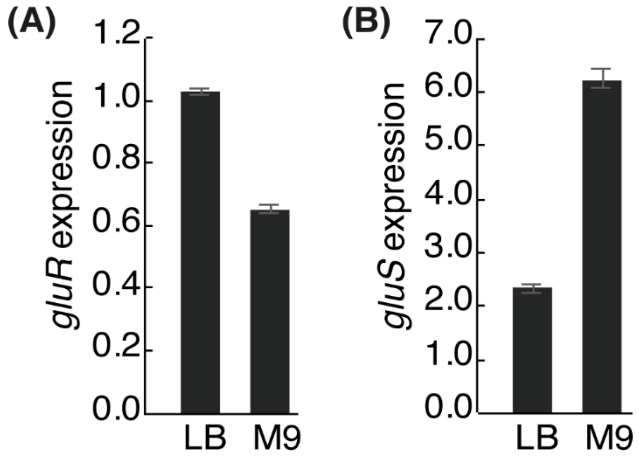

Figure S3. A mutation in the GluR response regulator did not affect the growth or survival of *B. glumae* at 37°C. Cell population densities of bacteria strains in LB medium were quantified in terms of colony-forming units (CFUs), and the results expressed as means (log CFU/ml) ± standard error (SE) of triplicate results.

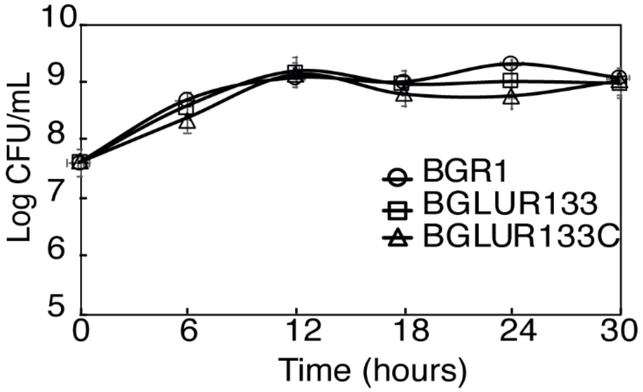

Figure S4. The upstream sequences of *ftsA* and *ftsZ* that were used in EMSA. The sequences with red underlining are conserved inverted repeat sequences, while those with black underlining are possible ribosome binding sites within the sequences. The inverted repeat sequences are indicated by the arrows with the conserved regions in both genes highlighted.

ftsA promoter regions

CGCTGGAGGAGGTCACGCTGTCGTCGCGCTACGCCTGGACGGTCAAGCTGGCAAACGGC
CTGGAGATCGAGTTCGGGCGCGAGCGCAACGCGGACACGCTGCCCGATCGCGCGCAAC
GGCTGGTGGCGGCGTGGCCGGCCGTCACCCAGCGCTGGGGGGCCGACATCGAGTACGC
GGACCTGCGCTATCCGAACGGGTTCGCGATTCGCGCGGCAGGCATGCGCTTTCTGAGCG
ACACCGACCACGGCAAGAAGTAACAGGACATCACACGCAAGAGCACTT**ATG**

ftsZ promoter regions

GCGGGCGGTCTGGCCGACGTGGTGCGCAATCCGCGCTACTCGACGGCGATGGGCCTGCT
CGTGGAAGGCAGCGCGCAACGAATGCGCGGCCGCAAGGTCGCAGTGCAATCGGGTTCG
GCGGGCCAGATCTTCACGCGGATGAAGGACTGGTTCCTGAGCAATTTCTGAACGGGTTT
TGCGCCGGTGCCGGCGGCCGGCGCGCGACGGGAGGTTGCCCGATCTTCCGCCGGATAAC
GGCCGAGTGGTTATATCTTTCTTGACGGAGGCAACA**ATG**

B. glumae BGR1 *ftsA* CGCGGACACGCTGCCCG
B. glumae BGR1 *ftsZ* CGCGCAACGAATGCGCG

Figure S5. Glutamate utilization of *B. glumae* BGR1 in LB culture medium over time. Data represents the mean ± standard deviation (SD) of triplicates.

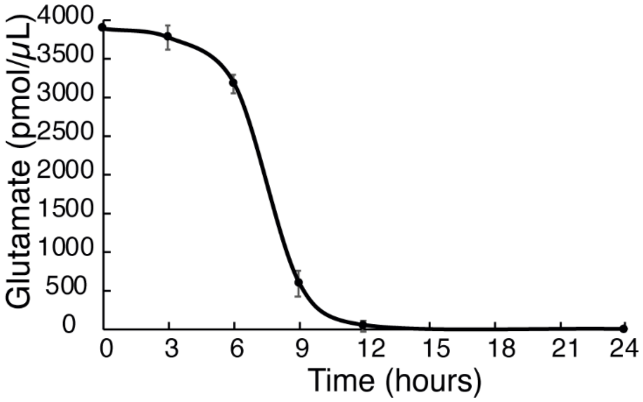

Figure S6. Electrophoresis gel image showing that *gluS* and *gluR* genes are co-transcribed in BGR1. A 237 bp product encompassing *gluR* and *gluS* genes was amplified by RT-PCR. The gel image was visualized using ChemiDoc XRS+ and Image Lab Software (Bio-Rad). Lane G, PCR product using genomic DNA as a template; Lane R, PCR product using RNA as a template; Lane C, PCR product using cDNA as a template.

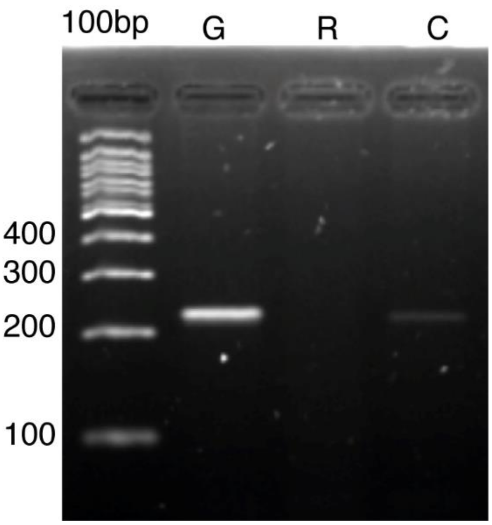

Figure S7. Electrophoresis gel image of RT-PCR showing no polar effect resulted from Tn*3*-*gusA* insertion. Lane bp, marker; Lane G1, PCR product from *gluR* chromosomal DNA as a template; Lane G2, PCR product from *gluS* chromosomal DNA as a template; Lane R, PCR product from total RNA as a template; Lane C1, PCR product from *gluR* cDNA as a template; Lane C2, PCR product from *gluS* cDNA as a template. All the experiments were performed in parallel. Gel images were visualized using ChemiDoc XRS+ and Image Lab Software (Bio-Rad).

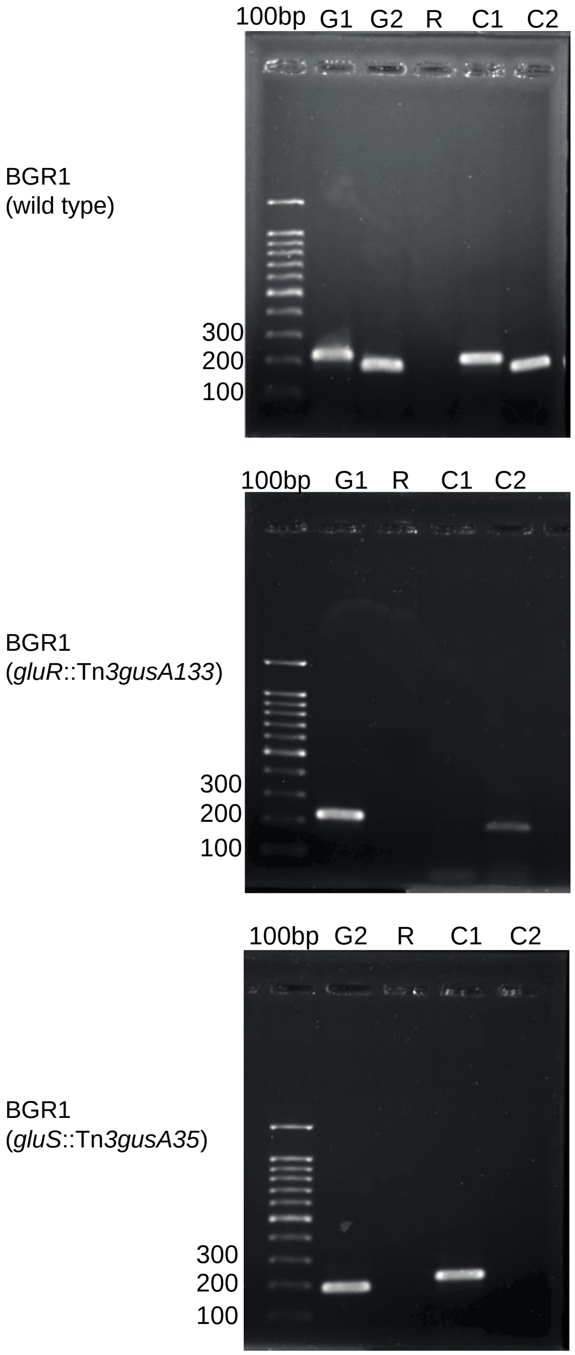

Figure S8. Electrophoretic mobility shift assay (EMSA) showing direct control of *ftsA* and *ftsZ* by GluR-His binding to the respective putative promoter regions. **(A - C)** Represents multiple exposures with different contrasts of the same respective blot.

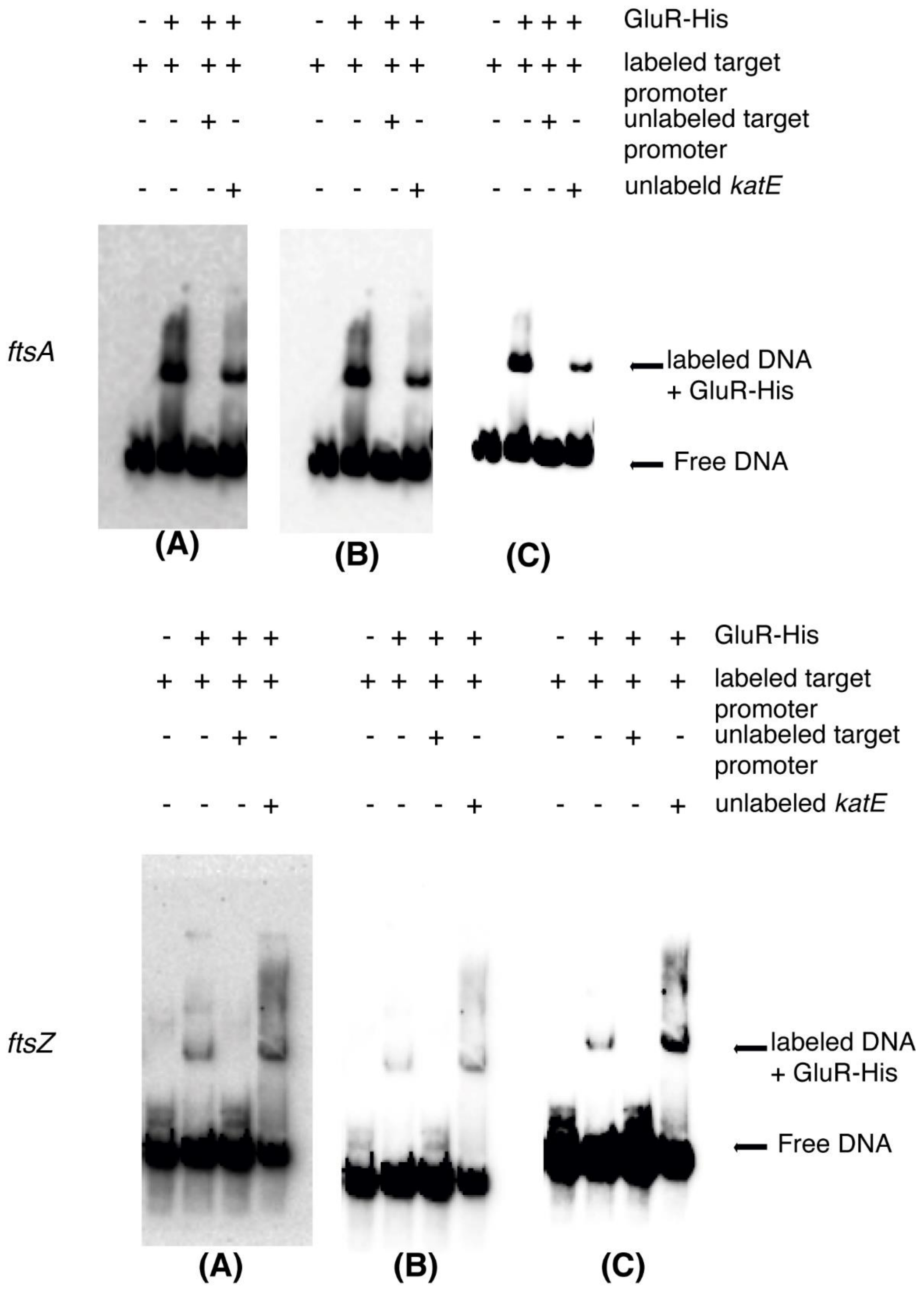

Table S1. Bacteria strains used in this study.

Strain or plasmid	Characteristics	Source
Burkholderia glumae		
BGR1	Wild type, Rif^R	Kim et al., 2004
BGLUR133	BGR1 *gluR*::Tn*3-gusA133*, Km^R	This study
BGLUS35	BGR1 *gluS*::Tn*3-gusA35*, Km^R	This study
BGLUR133C	BGR1, *gluR*::Tn*3-gusA133* containing pBGH13, Km^R, Tet^R	This study
RT271	BGR1 *gluR*::mini-Tn*5rescue*, Km^R	This study
RT271C	BGR1 *gluR*::mini-Tn*5rescue* containing pBGH1, Km^R, Tet^R	This study
Escherichia coli		
DH5α	F^- Φ80d*lacZ ΔM15*(*lacZYA-argF*) U169 *recA1 endA1 hsdR17* ($r_k^+m_k^+$ *supE44 thi-1 gyrA relA1*	Gibco BRL
BL21 (DE3)	F^- *ompT* $hsdS_B$ ($rB^-m_B^-$) *gal dcm* (DE3)	Novagen
Plasmid		
pBluescript II SK (+)	Cloning vehicle; phagemid, pUC derivative, Amp^R	Stratagene
pRK2013	Tra^+, ColE1 replicon, Km^R	Figurski and Helinski, 1979
pLAFR3	Tra^-, Mob^+ RK2 replicon, Tet^R	Staskawicz et al., 1987
pBGH1	13 kb BGR1 library clone containing *gluS* and *gluR* cloned into pLAFR3, Tet^R	This study
pBGH13	6.3 kb *Bam*H1-*Hin*dIII fragment containing *gluS* and *gluR* gene cloned into pLAFR3, Tet^R	This study
pET21b	T7 promoter-based expression vector, Amp^R	Novagen
pGluR-His	*gluR* in pRT21b, Amp^R	This study

Rif^R, rifampicin resistance; Tet^R, tetracycline resistance; Km^R, Kanamycin resistance; Amp^R, ampicillin resistance; Sp^R, Spectinomycin resistance.

Table S2. List of primers used in this study.

Primers	Sequence (5' to 3')
GluSR-F	CAGCCCGCGCTTCATTCAGA
GluSR-R	TGACCGCGATCAGCAGCGCG
FtsA-F	CTGCAAGATCACCAACGTTT
FtsA-R	TCGGTCTGCGTGACCTCCTT
FtsB-F	AGGATTTGCAGAACGGCACC
FtsB-R	ATTCGGCGAAACGAAACTGC
FtsI-F	ACATCACCTACGCCAACCRG
FtsI-R	CGGGTAATTGACGAGCGAGA
FtsK-F	GCATGTGGTCGCTGAAGGT
FtsK-R	GGAAGTACAGCGACAGACCG
FtsL-F	ATCTTCTTCCAGTTGCAGCGT
FtsL-R	CTCGATGCGCGAGGTCTT
FtsQ-F	AGGGCAACTTCTTCACGGTC
FtsQ-R	GAGCGGCTTGTATTCCTCCA
FtsW-F	CGTGGGACGAGCGCTATGCG
FtsW-R	TAGTTGAGCTTCTCGACGCT
FtsZ-F	ATGCCGAGATGGACAAGTGC
FtsZ-R	TCGAAGTCGACGTTAACGAG
16S RNA-F	AGCCGCGGTAATACGTAGG
16S RNA-R	ACTCTAGCCTGCCAGTCACC
KatE1-F	ACTCGCGCCGCTCGTCGAA
KatE1-R	ACATCGGCATCCTGGGTCGC
gluR_Nde1-F	GGCATATGATGCCGCCCATGGAAACTAA

gluR_BamH1-R	CCGGATCCGTCCGGGATGAAGACGTAGC
ftsAp- F	CGTCGCGCTACGCCTGGACG
ftsAp- R	TGTCGCTCAGAAAGCGCATG
ftsZp- F	TCGACGGCGATGGGCCTGCT
ftsZp- R	AAAGATATAACCACTCGGCC

F, forward; R, reverse.

SUPPLEMENTAL REFERENCES

Figurski, D.H., and Helinski, D.R. (1979). Replication of an origin-containing derivative of plasmid RK2 dependent on a plasmid function provided in trans. *Proc. Natl. Acad. Sci. U. S. A.* 76**,** 1648-1652.

Kim, J., Kim, J.G., Kang, Y., Jang, J.Y., Jog, G.J., Lim, J.Y., et. al. (2004). Quorum sensing and the LysR-type transcriptional activator ToxR regulate toxoflavin biosynthesis and transport in *Burkholderia glumae*. *Mol. Microbiol.* 54**,** 921-934. doi: 10.1111/j.1365-2958.2004.04338.x

Staskawicz, B., Dahlbeck, D., Keen, N., and Napoli, C. (1987). Molecular characterization of cloned avirulence genes from race 0 and race 1 of *Pseudomonas syringae* pv. *glycinea*. *J. Bacteriol.* 169**,** 5789-5794.
